# Supplementary material for: Cytogenetic Identification and Molecular Marker Analysis of Two Wheat–Thinopyrum ponticum Translocations with Stripe Rust Resistance
Source: Plants (Basel). 2024 Dec 25;14(1):27. doi: 10.3390/plants14010027 (PMC11723386; doi:10.3390/plants14010027)
Supplement: Supplementary file 1 [file plants-14-00027-s001.zip › plants-3294401-supplementary.pdf]

Table S1. Information on five specific intron-targeting markers of SN21171 and SN52684.

Marker Name	Primer Sequence (5′–3′)
CINAU1185F	GGCGACTCACACTTCTTGAA
CINAU1185R	CGAACAAGCTGCTCATATAATGG
CINAU1195F	TCAGGAGAGGTTGCTTCAGA
CINAU1195R	ATTTGCCCTTTGGTGTTGGC
CINAU1214F	TGATGCTCAAACTTCATGATGTT
CINAU1214R	ATTCAGCAGCTACGACACCA
CINAU957F	TTGCCTTCTACAACATTCCGA
CINAU957R	TACCAATCTCTGCTGTCCCC
CINAU959F	TCATCTGCCGAGGATCAATAGT
CINAU959R	TGTAGGCGTATCAGTTGGCT
